# Supplementary figures and images for: Stability of an ophthalmic formulation of polyhexamethylene biguanide in gamma-sterilized and ethylene oxide sterilized low density polyethylene multidose eyedroppers
Source: PeerJ. 2018 Apr 18;6:e4549. doi: 10.7717/peerj.4549 (PMC5910790; doi:10.7717/peerj.4549)

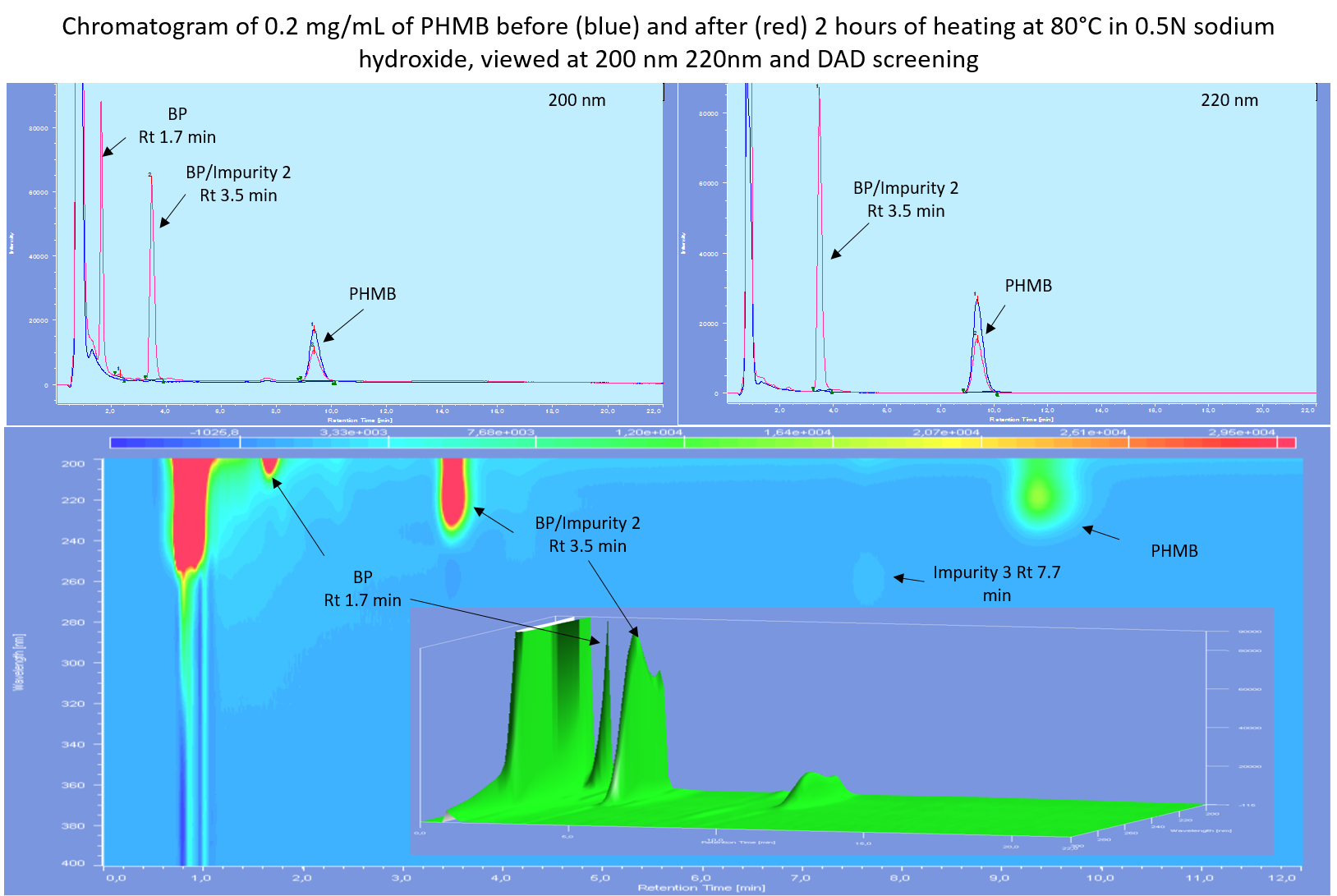

Supplement: Supplemental Information 6 [file peerj-06-4549-s006.png]

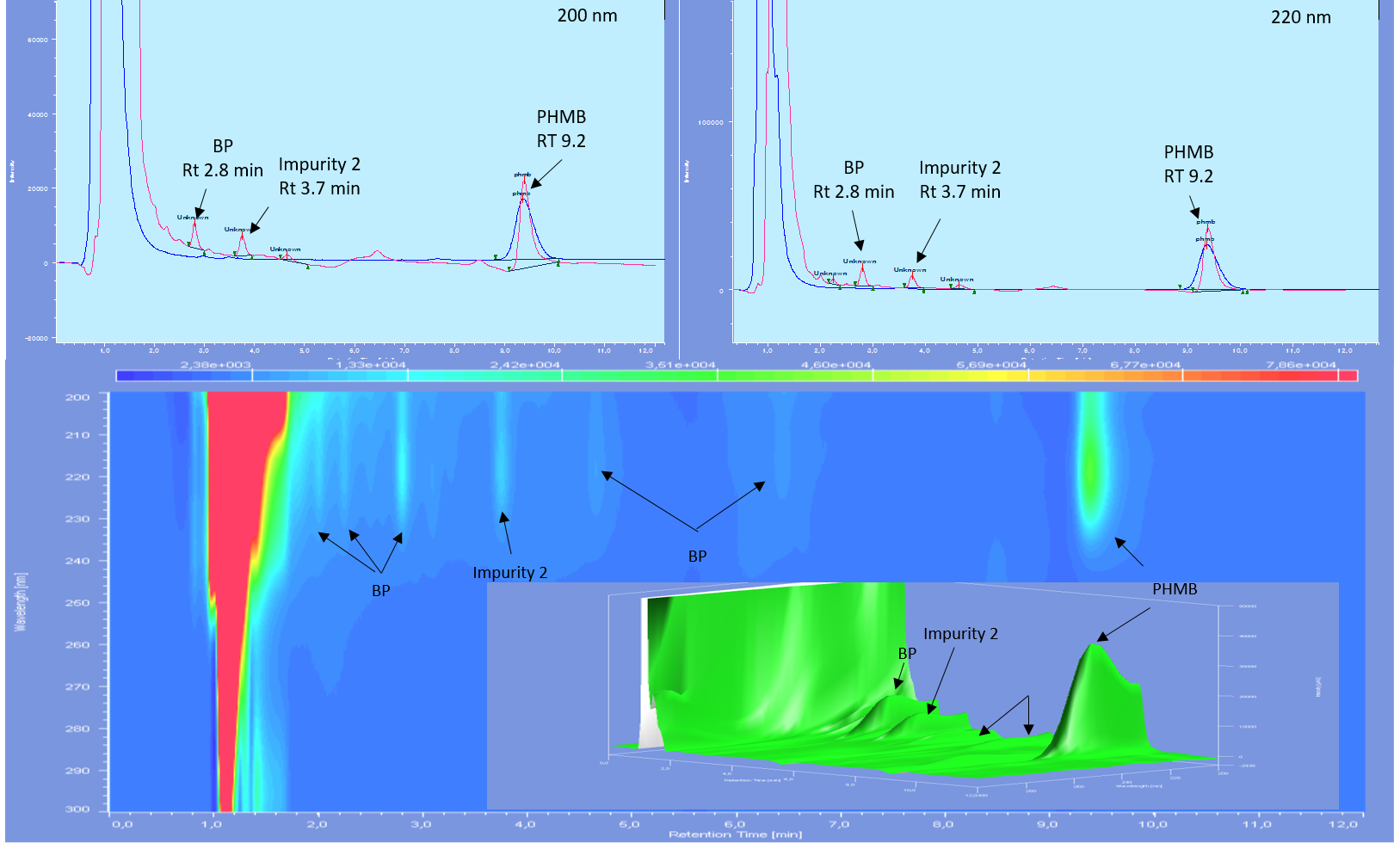

Supplement: Supplemental Information 7 [file peerj-06-4549-s007.png]

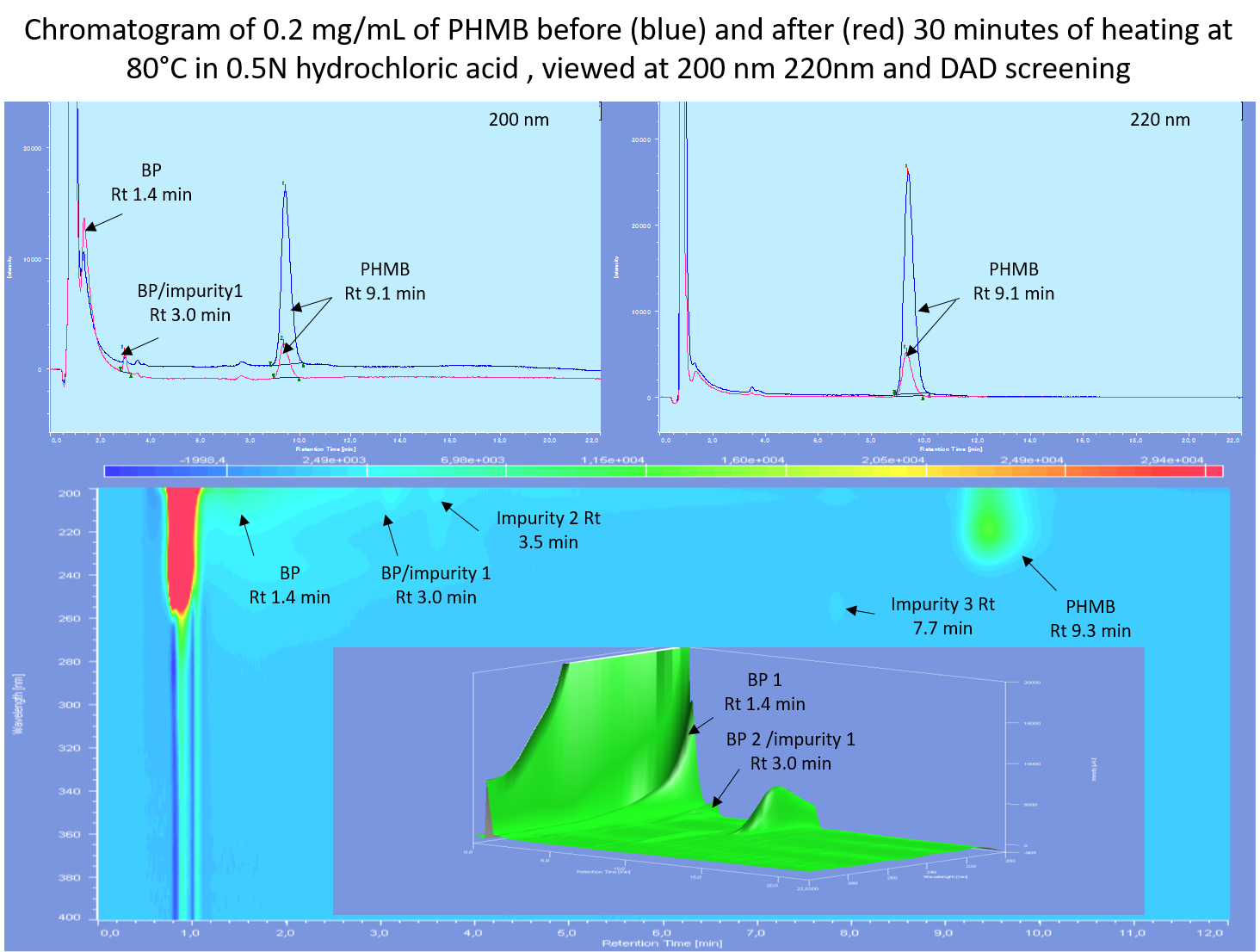

Supplement: Supplemental Information 8 [file peerj-06-4549-s008.png]

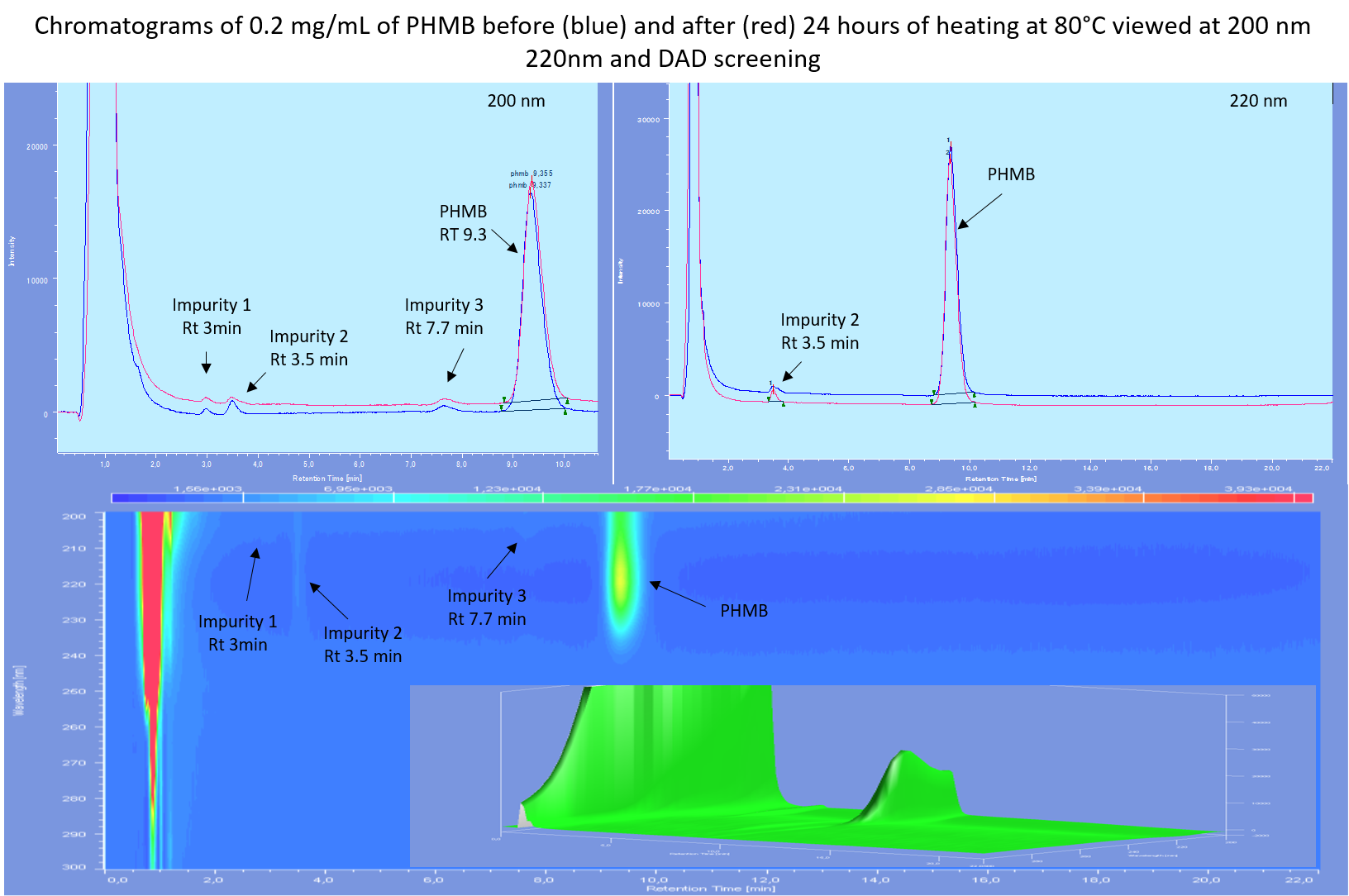

Supplement: Supplemental Information 9 [file peerj-06-4549-s009.png]
